# Time course of the triglyceride glucose index accumulation with the risk of cardiovascular disease and all-cause mortality

**DOI:** 10.1186/s12933-022-01617-2

**Published:** 2022-09-13

**Authors:** Xue Tian, Shuohua Chen, Yijun Zhang, Xiaoli Zhang, Qin Xu, Penglian Wang, Shouling Wu, Anxin Wang, Yanxia Luo

**Affiliations:** 1 Department of Epidemiology and Health Statistics, School of Public Health, Capital Medical University, No.10 Xitoutiao, You’anmen Wai, Fengtai District, 100069 Beijing, China; 2 Beijing Municipal Key Laboratory of Clinical Epidemiology, Beijing, China; 3 Department of Cardiology, Kailuan Hospital, North China University of Science and Technology, 57 Xinhua East Rd, 063000 Tangshan, China; 4 Department of Neurology, Beijing Tiantan Hospital, Capital Medical University, Beijing, China; 5 Beijing Tiantan Hospital, China National Clinical Research Center for Neurological Diseases, Capital Medical University, No.119 South 4th Ring West Road, Fengtai District, 100070 Beijing, China

**Keywords:** Cardiovascular disease, All-cause mortality, Triglyceride-glucose index, Time course analysis, Cohort study

## Abstract

**Background:**

Future risk of cardiovascular disease (CVD) and mortality is associated with cumulative amount TyG index (cumTyG) exposure, while whether time course of TyG accumulation modulates the risk remains unclear. This study sought to examine the associations of cumTyG index accumulation time course with the risk of CVD and all-cause mortality.

**Methods:**

We enrolled 51,734 participants free of CVD and underwent three examinations at year 2006, 2008, and 2010. CumTyG from baseline to the third examination was calculated. Time course of cumTyG accumulation was calculated as the slope of TyG versus time from 2006 to 2010, or as splinting the overall TyG index accumulation into early (cumTyG_06 − 08_) and late accumulation (cumTyG_08 − 10_). Participants were categorized by the combination of cumTyG < or ≥ median (34.44 × years) and a negative or positive TyG slope.

**Results:**

During a median follow-up of 9.04 years, we identified 3,602 incident CVD cases and 3,165 deaths. The risk of CVD and all-cause mortality increased with decreased TyG slope, the corresponding adjusted hazard ratio (aHR) with 95% confidence interval (CI) was 1.11 (1.04–1.19) and 1.18 (1.10–1.26) for patients with a negative TyG slope, respectively. Consistently, a later accumulation of TyG index was not associated with the risk of CVD and all-cause mortality after adjustment for an early accumulation. When considering the combination of cumTyG index and time course, participants with a cumTyG ≥ median and a negative TyG slope had elevated risk of CVD (aHR, 1.37; 95% CI, 1.24–1.51) and all-cause mortality (aHR, 1.28; 95% CI, 1.15–1.43). Additionally, the association was more prominent in young adults.

**Conclusion:**

Early TyG index accumulation resulted in a greater risk of CVD and all-cause mortality than later TyG later accumulation with the same overall cumulative exposure, emphasizing the importance of optimal TyG index control earlier in life.

**Supplementary Information:**

The online version contains supplementary material available at 10.1186/s12933-022-01617-2.

## Background

Insulin resistance (IR) is one of the most important risk factors for cardiovascular disease (CVD) [1]. IR is associated with obesity [2], hypertension [3], diabetes [4], and dyslipidemia [5], all of these predispose individuals to atherosclerosis and CVD. Indeed, the associations between IR and CVD risk have been well-documented in previous studies [6–8]. Many recent investigations have shown that the triglyceride glucose (TyG) index, which is derived from fasting triglyceride (TG) and fasting blood glucose (FBG), has high correlation with IR assessed by hyperinsulinemic euglycemic clamp testing and the homeostasis model assessment of IR (HOMA-IR) [9–11]. Thus, the TyG index has been proposed as a simple, cost-effective, reproducible, and reliable surrogate marker for IR.

Consistent with these data, there is growing evidence to suggest that not only baseline TyG index, but also long-term cumulative amount of TyG index (cumTyG) exposure is associated with the risk of CVD events [1, 12–16]. Elevated TyG index confers risk for CVD that increases with increasing TyG index concentrations and longer duration of exposure. Incorporation both the TyG index concentration and exposure duration into a single risk parameter for future CVD is intuitively appealing, although a data-based demonstration of the utility of this metric is not available. Also unclear is whether the time course of TyG index accumulation is important in modulating the risk conferred by a given exposure amount. For instance, does the risk of CVD in an individual subsequent to a particular total amount of TyG index over a similar exposure duration differed by accumulation at earlier and later?

Therefore, this study sought to examine the associations between time course of the TyG index accumulation with the risk of CVD and all-cause mortality in a large community-based Chinese cohort.

## Methods

### Study population

Data were obtained from the Kailuan study, which is an ongoing prospective cohort study conducted in Tangshan, China. The details of the study design and procedures have been described previously [17–19]. During July 2006 to October 2007, a total of 101,510 adults (81 110 men and 20 400 women, aged 18–98 years) were enrolled to participate in the baseline questionnaire interview, clinical and laboratory examinations at 11 hospitals affiliated with the Kailuan Group. All the participants were followed up biennially until the date of their death or 31 December 2019. In the current analysis, we excluded 44,677 participants with less than 3 health examinations at year 2006, 2008, and 2010, 1,034 participants with missing data on TG or FBG, and 4,065 participants who had CVD or died in or prior 2010. Terminally, a total of 51,734 participants were included for the analysis of the time course of TyG accumulation and the risk of subsequent CVD (Additional file 1: Figure S1). The study was performed according to the guidelines of the Declaration of Helsinki and was approved by the Ethics Committee of Kailuan General Hospital and Beijing Tiantan Hospital. All participants provided written informed consent.

### Data collection

Information on demographic characteristics (age, sex), lifestyle (physical activity, smoking, drinking status), and medical history (hypertension, diabetes, dyslipidemia, and use of antihypertensive, antidiabetic, and lipid-lowering agents) was collected through face-to-face interviews via a standard questionnaire. Body mass index (BMI) was calculated by dividing body weight (kg) by the square of height (m^2^). Blood pressure was measured in the left upper arm using a calibrated mercury sphygmomanometer with the participants in a sitting position. At least 2 blood pressure measurements were taken after 5 min of rest. Blood pressure was then measured again if the difference between the 2 measurements was ≥ 5 mm Hg. The average was used as systolic blood pressure (SBP) and diastolic blood pressure (DBP).

Fasting blood samples were collected from the antecubital vein after an 8- to 12- h overnight fast. All the plasma samples were assessed using an auto-analyzer (Hitachi 747, Tokyo, Japan) at the central laboratory of Kailuan Hospital. FBG levels were measured using the hexokinase/glucose-6-phosphate dehydrogenase method with the coefficient of variation using blind quality control specimens < 2.0%. Serum total cholesterol (TC), TG, low-density lipoprotein cholesterol, and high-density lipoprotein cholesterol levels were measured with the enzymatic colorimetric method. Estimated glomerular filtration rate (eGFR) was calculated used Chronic Kidney Disease Epidemiology Collaboration creatinine equation [20]. High-sensitivity C-reactive protein (hs-CRP) levels were measured with high-sensitivity particle-enhanced immunonephelometry assay.

Hypertension was defined as SBP ≥ 140 mmHg or DBP ≥ 90 mmHg, or with a self-reported history of physician-diagnosed hypertension, or use of antihypertensive drugs. Diabetes mellitus was defined as any self-reported diabetes mellitus or use of glucose-lowering drugs, or FBG ≥ 7 mmol/L. Dyslipidemia was defined as any self-reported history or use of lipid-lowering drugs, or serum TC ≥ 5.17 mmol/L or triglyceride ≥ 1.69 mmol/L or low-density lipoprotein cholesterol ≥ 3.62 mmol/L or high-density lipoprotein cholesterol ≤ 1.04 mmol/L.

### Calculation on time of TyG index accumulation

The TyG index was calculated as ln [(fasting TG (mg/dl)×FBG (mg/dl)/2] [21–23], and cumTyG index was defined as the summed average TyG index for each pair of consecutive examinations multiplied by the time interval between two consecutive examinations in years, as described previously [24, 25] (Additional file 1: Figure S2).

Time course of the TyG index accumulation was categorized in two ways: a slope of the TyG index over time from 2006 to 2010 using the linear regression and the least-squares principle, where the TyG index was taken as the dependent variable, and time as the independent variable, with a positive or negative slope indicating an increase or decrease in the TyG index over time (Additional file 1: Figure S2); or alternatively, the cumTyG from 2006 to 2008 and from 2008 to 2010 were calculated as the early and late TyG index exposure measure, respectively. Furthermore, the overall TyG index accumulation and its time course were combined into one metric.

### Outcome assessment

The outcomes in the present study were the first occurrence of CVD or all-cause mortality. The types of CVD included stroke (ischemic and hemorrhagic stroke) and myocardial infarction (MI), heart failure, and atrial fibrillation. All participants were linked to the Municipal Social Insurance Institution and the Hospital Discharge Register for incidence of CVD, which cover all of the Kailuan study participants. To further identify potential CVD events, we reviewed the discharge lists from the 11 hospitals during 2006–2019 and asked for a history of CVD via a questionnaire during the biennial interview. For all suspected CVD events, 3 experienced physician adjudicators who were blinded to the study design reviewed the medical records. Incident stroke was diagnosed based on neurological signs, clinical symptoms, and neuroimaging tests, including computed tomography or magnetic resonance, according to the World Health Organization criteria [26]. MI was diagnosed according to the criteria of the World Health Organization on the based on the clinical symptoms, changes in the serum concentrations of cardiac enzymes and biomarkers, and electrocardiographic results [27]. Heart failure was defined in accordance with the criteria of the European Society of Cardiology [28] on the basis of clinical symptoms, echocardiography, chest X-ray, and electrocardiography. Atrial fibrillation was ascertained based on 12-lead electrocardiograms according to the European Society of Cardiology guidelines [29]. All-cause mortality data were gathered from provincial vital statistics offices and reviewed by physicians.

### Statistical analysis

Participants were categorized by a positive (or negative) slope of TyG versus time relationship indicating either an increasing (or decreasing) trend over time, or the combination of the overall cumTyG (< or ≥ its median [34.44 × years]) with TyG index slope. Continuous variables were compared using student *t* test, analysis of variance, Wilcoxon, or the Kruskal-Wallis test accosting to distribution, and categorical variables were compared with the chi-square test. Incidence rate per 1000 person-years was calculated by dividing the confirming incident cases by the total person-years of follow-up and multiplying by 1000. The cumulative incidences of CVD and all-cause mortality were estimated using Kaplan-Meier curves and the differences in curves were compared using the log-rank test.

The combination of the overall cumTyG exposure and TyG slope was the primary exposure in our study. We performed progressively adjusted Cox proportional hazard regressions to assess the associations of TyG accumulation time course with the risk of CVD and all-cause moratlity, with cumTyG < median and TyG slope ≥ 0 as the reference group. Model 1 was unadjusted; model 2 was adjusted for age and sex; model 3 was further adjusted for education, income, physical activity, smoking status, drinking status, history of hypertension, diabetes, dyslipidemia, antihypertensive, antidiabetic, and lipid-lowering agents, BMI, SBP, DBP, TC, eGFR, and hs-CRP. To capture the dose–response relationships of TyG index slope with the risk of CVD and all-cause mortality, restricted cubic splines analysis was performed with three knots at the 10th, 50th, and 90th percentiles of TyG index slope distribution, with 0 as the reference point and the HRs were adjusted for variables in Model 3.

Additional analyses were performed to validate the robustness of the results. First, when CVD events as the outcome of interest, the competing risk model was applied with non-CVD death as a competing risk event. Whereas when all-cause mortality as the outcome, we excluded CVD events during the follow-up period. Second, to explore the potential impact of reverse causality, 2-year lagged analysis was performed by repeating the primary analysis and excluded participants who developed CVD cases or died within the first 2 years of follow-up. Third, participants who were treated with antidiabetic or lipid-lowering agents were excluded, considering these medications may have some impacts on the level of TyG index. Fourth, restricted analysis was performed by excluding participants with abnormal FBG (≥ 7.0 mmol/L) or TG (≥ 1.7 mmol/L) concentrations at baseline. Subgroup analyses were conducted on the participants after stratification by age (< 60 vs. ≥ 60 years), sex, diabetes history, and BMI (< 25 vs. ≥ 25 kg/m^2^), interactions between subgroups and cumTyG index time course were tested using likelihood ratio tests, in which modes with and without multiplicative interaction terms were compared.

All analyses were performed using SAS version 9.4 (SAS Institute, Cary, NC, USA). All the statistical tests were 2-sided, and *P* < 0.05 was considered statistical significance.

## Results

### Baseline characteristics

A total of 51,734 participants were enrolled in the current analysis, the differences of the baseline characteristics between included and excluded participants are provided in Additional file 1: Table S1. The mean age of the enrolled participants was 48.76 ± 11.77 years, and 39,387 participants (76.13%) were men, the mean cumTyG was 35.09 ± 4.72 ×years, and the mean slope was 0.01 per year. The number of participants with a negative TyG slope was 23,450, and 28,284 participants had a positive TyG slope. Compared with participants with a positive TyG slope, those with a negative TyG slope were more likely to have more cardiovascular risk factor profiles (Additional file 1: Table S2).

Baseline characteristics according to cumTyG and TyG slope are presented in Table 1. Participants with cumTyG ≥ median and a TyG slope < 0 were more likely to be older, men, had a higher prevalence of hypertension, diabetes, dyslipidemia, were more likely to take antihypertensive, antidiabetic, and lipid-lowering agents, and had a higher level of BMI, SBP, DBP, FBG, TC, LDL-C, hs-CRP, and a lower level of eGFR, compared with participants in other groups (Table 1).

**Table 1:** Baseline characteristics according to cumulative accumulation and slope of the TyG index

Characteristics	Overall (N = 51,734)	CumTyG < median, slope ≥ 0 (N = 14,340)	CumTyG < median, slope < 0 (N = 11,527)	CumTyG ≥ median, slope ≥ 0 (N = 13,944)	CumTyG ≥ median, slope < 0 (N = 11,923)	*P* value
Age, years	48.76 ± 11.77	44.79 ± 10.92	46.84 ± 11.00	51.01 ± 11.88	52.79 ± 11.44	< 0.0001
Men, n (%)	39,387 (76.13)	10,690 (74.55)	8787 (76.23)	10,597 (76.00)	9313 (78.11)	< 0.0001
High school or above, n (%)	4187 (8.38)	1189 (8.59)	772 (6.89)	1260 (9.40)	966 (8.38)	< 0.0001
Income ≥ 1000RMB, n (%)	7751 (15.53)	2090 (15.11)	1256 (11.22)	2433 (18.19)	1972 (17.12)	< 0.0001
Current smoker, n (%)	17,476 (34.72)	5030 (36.08)	3570 (31.62)	4899 (36.31)	3977 (34.25)	< 0.0001
Current alcohol, n (%)	20,243 (40.2)	5747 (41.22)	3957 (35.03)	5781 (42.84)	4758 (40.94)	< 0.0001
Active physical activity, n (%)	6979 (13.49)	1346 (9.39)	1067 (9.26)	2362 (16.94)	2204 (18.49)	< 0.0001
Hypertension, n (%)	19,650 (37.98)	4106 (28.63)	4373 (37.94)	5513 (39.54)	5658 (47.45)	< 0.0001
Diabetes mellitus, n (%)	4044 (7.82)	331 (2.31)	656 (5.69)	1154 (8.28)	1903 (15.96)	< 0.0001
Dyslipidemia, n (%)	17,472 (33.77)	2444 (17.04)	3304 (28.66)	5161 (37.01)	6563 (55.04)	< 0.0001
Antihypertensive agents, n (%)	4311 (8.33)	633 (4.41)	543 (4.71)	1634 (11.72)	1501 (12.59)	< 0.0001
Hypoglycemic agents, n (%)	953 (1.84)	84 (0.59)	93 (0.81)	348 (2.50)	428 (3.59)	< 0.0001
Lipid-lowering agents, n (%)	404 (0.78)	50 (0.35)	29 (0.25)	178 (1.28)	147 (1.23)	< 0.0001
Body mass index, kg/m^2^	25.04 ± 3.48	24.36 ± 3.42	24.76 ± 3.47	25.39 ± 3.43	25.73 ± 3.43	< 0.0001
SBP, mmHg	127.90 ± 19.64	123.44 ± 18.06	127.37 ± 19.53	129.13 ± 19.79	132.34 ± 20.22	< 0.0001
DBP, mmHg	82.37 ± 11.29	80.41 ± 10.82	82.68 ± 11.46	82.57 ± 11.23	84.2 ± 11.40	< 0.0001
FBG, mmol/L	5.38 ± 1.52	4.98 ± 0.85	5.31 ± 1.27	5.36 ± 1.37	5.96 ± 2.19	< 0.0001
Total cholesterol, mmol/L	4.91 ± 1.12	4.78 ± 0.94	4.79 ± 1.13	5.07 ± 1.08	5.02 ± 1.32	< 0.0001
Triglyceride, mmol/L	1.67 ± 1.37	1.01 ± 0.52	1.62 ± 1.10	1.64 ± 1.20	2.56 ± 1.91	< 0.0001
LDL cholesterol, mmol/L	2.29 ± 0.90	2.23 ± 0.88	2.29 ± 0.86	2.3 ± 0.92	2.34 ± 0.92	< 0.0001
HDL cholesterol, mmol/L	1.55 ± 0.39	1.55 ± 0.38	1.58 ± 0.38	1.52 ± 0.40	1.55 ± 0.41	< 0.0001
eGFR, mL/min/1.73 m^2^	84.31 ± 25.11	88.1 ± 24.42	82.45 ± 26.15	85.65 ± 25.57	80.00 ± 23.48	< 0.0001
hs-CRP, mg/L	2.30 ± 6.51	2.23 ± 5.75	2.06 ± 6.67	2.57 ± 8.11	2.28 ± 4.89	< 0.0001
CumTyG, ×years	35.09 ± 4.72	31.59 ± 2.01	31.65 ± 2.07	38.59 ± 3.90	38.52 ± 4.18	< 0.0001
Slope, year^− 1^	0.01 ± 0.16	0.11 ± 0.10	− 0.11 ± 0.09	0.13 ± 0.11	− 0.12 ± 0.11	< 0.0001

The median value of cumTyG was 34.44 × year

*cumTyG* cumulative TyG index,* DBP* diastolic blood pressure,* eGFR* estimated glomerular filtration rate,* FBG* fasting blood glucose,* hs-CRP* high-sensitivity C-reactive protein,* LDL* low density lipoprotein,* HDL* high density lipoprotein,* SBP* systolic blood pressure,* TyG index* triglyceride glucose index

### TyG accumulation time course

**Fig. 1:**
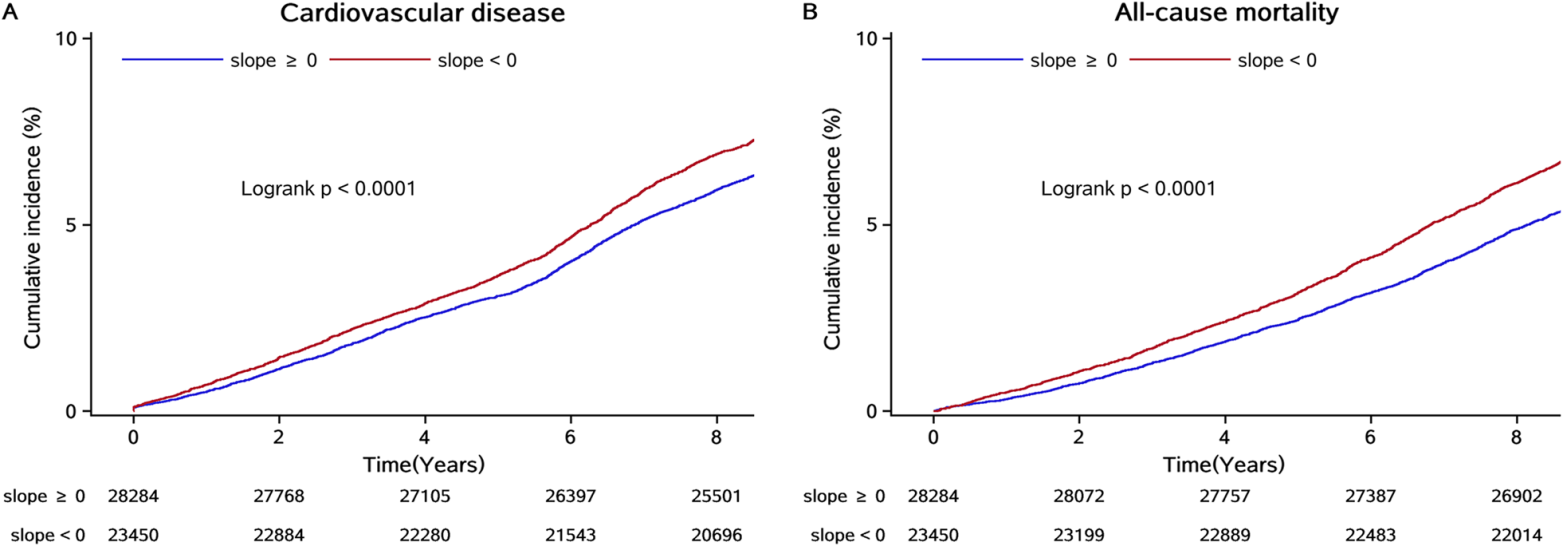
Kaplan-Meier curve of cardiovascular disease and all-cause mortality incidence rate by the time course of TyG index accumulation.* TyG index* triglyceride glucose index

**Fig. 2:**
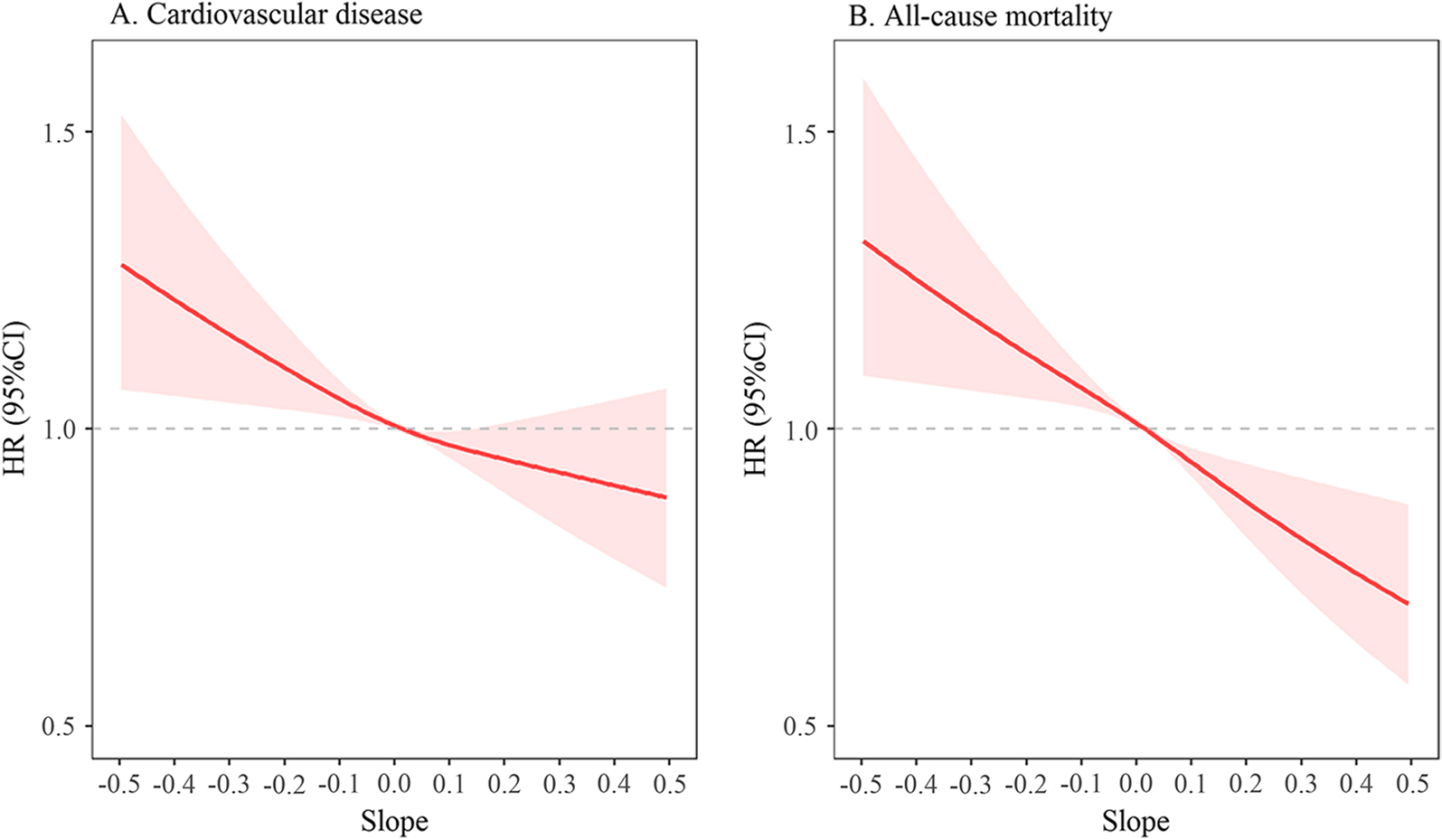
Hazard ratios and 95% CIs for the time course of TyG index accumulation with risk of cardiovascular disease and all-cause mortality by using restricted cubic spline regression with 5 knots with placed at the 5th, 25th, 50th, 75th, and 95th percentiles of the TyG index time course.* CI* confidence interval,* TyG index* triglyceride 725 glucose index. Adjusted for age, sex, education, income, drinking status, smoking status, physical activity, history of hypertension, diabetes, dyslipidemia, antihypertensive agents, antidiabetic agents, lipid-lowering agents, body mass index, systolic blood pressure, diastolic blood pressure, fasting blood glucose, total cholesterol, estimated glomerular filtration rate, and high sensitivity C-reactive protein

During a median follow-up of 9.04 years (interquartile range: 8.67–9.32 years), we identified 3,602 cases of incident CVD (including 2240 cases of stroke, 544 cases of MI, 774 cases of heart failure, and 321 cases of atrial fibrillation) and 3,165 cases of all-cause mortality. The associations of the time course of TyG index accumulation evaluated by adding the slope of TyG index over 2006–2010 to the analysis, or by splitting the overall accumulation into an early (cumTyG_06 − 08_) and late accumulation (cumTyG_08 − 10_) in the models are presented in Table 2. The incidence rate per 1000 person-years of CVD (8.86; 95% CI, 8.46–9.29) and all-cause mortality (7.85; 95% CI, 7.48–8.25) was higher in participants with a negative slope of TyG time course (decreasing trend) than that in participants with a positive slope of TyG time course (increasing trend) (Table 2; Fig. 1). After adjusted for potential variables, the risk of CVD and all-cause mortality increased with decreased TyG slope, showing a reverse J-shaped association in the multivariable-adjusted spline regression models (Fig. 2). Participants with a negative slope of TyG time course had a 1.11-fold higher risk of CVD (HR, 1.11; 95% CI, 1.04–1.19), and a 1.18-fold higher risk of all-cause mortality (HR, 1.18; 95% CI, 1.10–1.26), compared with participants with positive slope of TyG time course. Consistently, the later accumulation was not associated with the risk of CVD (HR, 1.02; 95% CI, 0.96–1.02; *P* = 0.3251) and all-cause mortality (HR, 1.03; 95% CI, 0.97–1.05; *P* = 0.4097) after adjusted for early accumulation in the multivariable models. Similar results were observed for CVD subtypes (Additional file 1: Table S3).

**Table 2:** Association of time course of cumTyG accumulation with risk of CVD and all-cause morality

	Slope^a^			cumTyG06-08		cumTyG08-10^b^	
	< 0	≥ 0	*P* value	HR (95% CI)	*P* value	HR (95% CI)	*P* value
CVD							
Cases, n (%)	1757 (7.49)	1845 (6.52)					
Incidence rate per 1000 person-years	8.86(8.46–9.29)	7.64(7.30–7.99)					
Model 1	1.16(1.09–1.24)	Reference	< 0.0001	1.05(1.04–1.06)	< 0.0001	1.03(0.98–1.05)	0.1078
Model 2	1.15(1.08–1.23)	Reference	< 0.0001	1.05(1.05–1.06)	< 0.0001	1.03(0.98–1.04)	0.1120
Model 3	1.11(1.04–1.19)	Reference	0.0014	1.04(1.03–1.04)	< 0.0001	1.02(0.96–1.03)	0.3251
All-cause mortality							
Cases, n (%)	1607 (6.85)	1558 (5.51)					
Incidence rate per 1000 person-years	7.85(7.48–8.25)	6.28(5.97–6.59)					
Model 1	1.25(1.17–1.34)	Reference	< 0.0001	1.07(1.07–1.08)	< 0.0001	1.05(0.99–1.08)	0.0864
Model 2	1.24(1.15–1.33)	Reference	< 0.0001	1.07(1.06–1.08)	< 0.0001	1.05(0.98–1.08)	0.1052
Model 3	1.18(1.10–1.26)	Reference	< 0.0001	1.06(1.05–1.07)	< 0.0001	1.03(0.97–1.05)	0.4097

Model 1: unadjusted

Model 2: adjusted for age and sex

Model 3: further adjusted for education, income, physical activity, smoking status, drinking status, history of hypertension, diabetes, dyslipidemia, antihypertensive agents, antidiabetic agents, lipid-lowering agents, body mass index, systolic blood pressure, diastolic blood pressure, total cholesterol, estimated glomerular filtration rate, and high sensitivity C reactive protein

*CI* confidence interval,* CVD* cardiovascular disease,* cumTyG* cumulative triglyceride glucose index,* HR* hazard ratio

^a^ further adjusted for mean TyG during 2006–2010

^b^ further adjusted for cumulative TyG during 2006–2008

### Combination of cumTyG and TyG slope

**Fig. 3:**
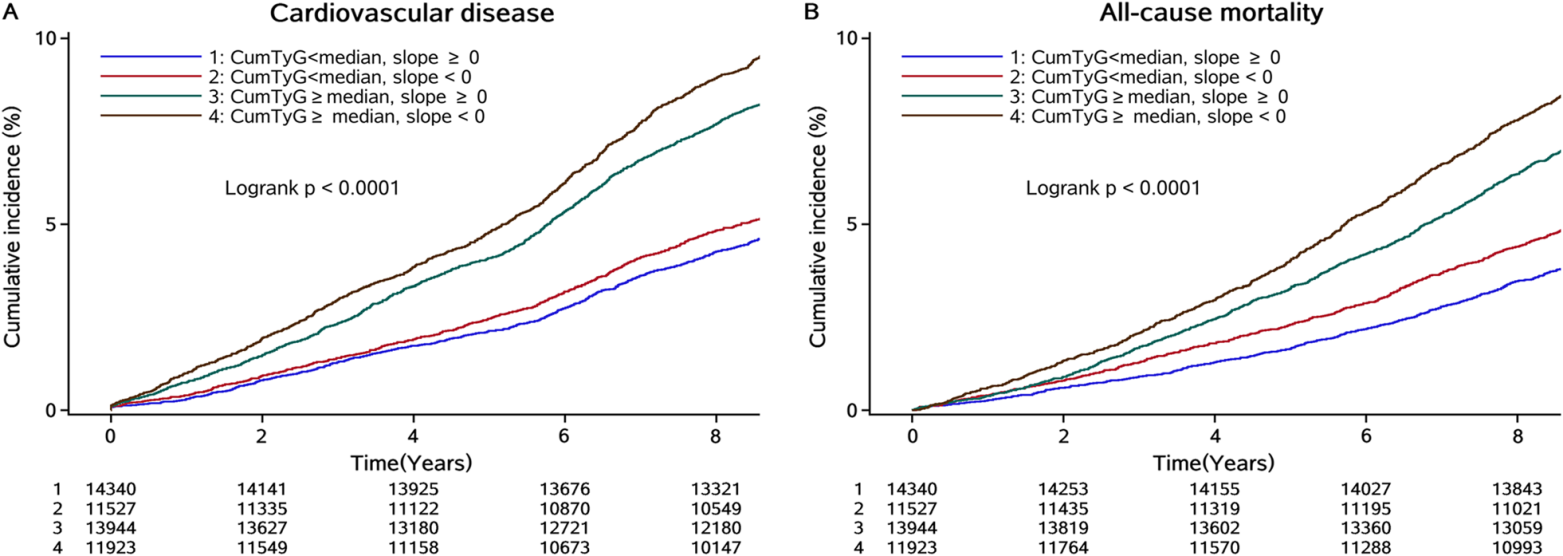
Kaplan-Meier curve of cardiovascular disease and all-cause mortality incidence rate by the combination of cumTyG and time course of TyG index accumulation.* cumTyG* cumulative triglyceride glucose index,* TyG index* triglyceride glucose index

**Fig. 4:**
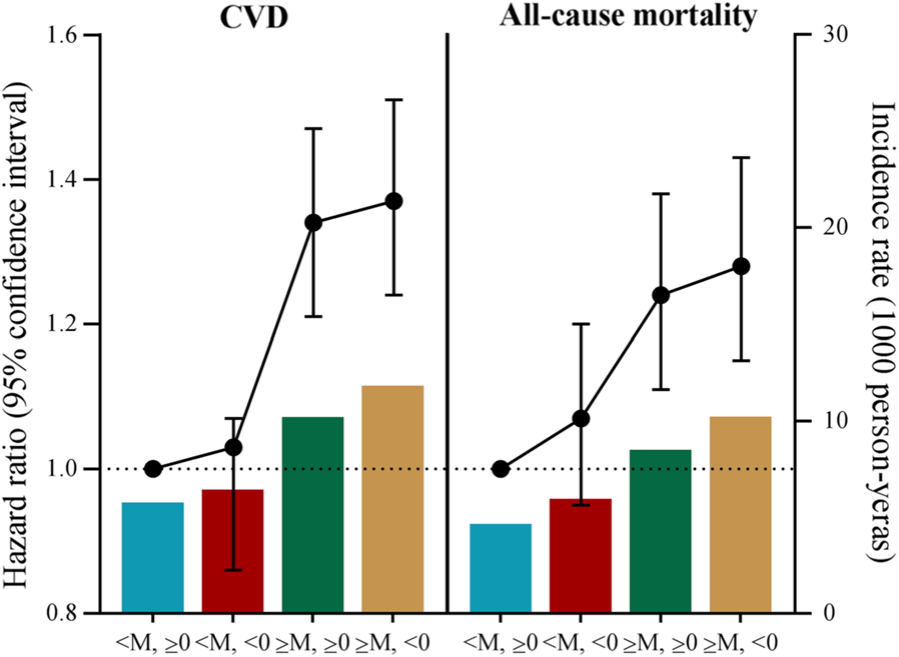
Incidence rate and hazard ratio of cardiovascular disease and all-cause mortality incidence rate by the combination of cumTyG and time course of TyG index accumulation. *cumTyG* cumulative triglyceride glucose index,* TyG index* triglyceride glucose index. The four categories of combination of cumTyG and TyG slope were as follows: <M, ≥ 0: cumTyG < Median (34.44 × years), and TyG slope ≥ 0; <M, < 0: cumTyG < Median, and TyG slope < 0; ≥M, ≥ 0: cumTyG ≥ Median, and TyG slope ≥ 0; ≥M, < 0: cumTyG ≥ Median, and TyG slope  < 0. Adjusted for age, sex, education, income, drinking status, smoking status, physical activity, history of hypertension, diabetes, dyslipidemia, antihypertensive agents, antidiabetic agents, lipid-lowering agents, body mass index, systolic blood pressure, diastolic blood pressure, fasting blood glucose, total cholesterol, estimated glomerular filtration rate, and high sensitivity C-reactive protein

When considering the combined effect of cumTyG accumulation and its time course, the incidence rate of CVD and all-cause mortality increased with elevated cumTyG and decreased TyG slope, reaching to 11.60 (95% CI, 11.00-12.30) and 10.00 (95% CI, 9.41–10.60) in the group of cumTyG ≥ median and a TyG slope < 0, respectively (Table 3; Fig. 2). In the fully adjusted model, the highest risk of CVD (HR, 1.37; 95% CI, 1.24–1.51) and all-cause mortality (HR, 1.28; 95% CI, 1.15–1.43) was observed for participants with cumTyG ≥ median and a TyG slope < 0, followed by those with cumTyG ≥ median and a TyG slope ≥ 0. The corresponding HR was 1.34 (95% CI, 1.21–1.47) for CVD, and 1.24 (95% CI, 1.11–1.38) for all-cause mortality, respectively, compared with those with cumTyG < 0 and a TyG slope ≥ 0. While the associations attenuated to a non-significant level in participants with cumTyG < median and a TyG slope < 0 (Table 3; Figs. 3 and 4).

**Table 3:** Associations of cumulative accumulation and slope of the TyG index with CVD and all-cause mortality

Outcomes	CumTyG < median, slope ≥ 0	CumTyG < median, slope < 0	CumTyG ≥ median, slope ≥ 0	CumTyG ≥ median, slope < 0	*P* for trend
CVD					
Cases, n (%)	701 (4.89)	629 (5.46)	1144 (8.20)	1128 (9.46)	
Incidence rate per 1000 person-years	5.52 (5.13–5.95)	6.22 (5.75–6.73)	9.98 (9.42–10.60)	11.60 (11.00-12.30)	
Model 1	Reference	1.13 (1.01–1.26)	1.82 (1.66-2.00)	2.13 (1.93–2.34)	< 0.0001
Model 2	Reference	1.07 (0.96–1.19)	1.53 (1.39–1.68)	1.67 (1.52–1.84)	< 0.0001
Model 3	Reference	1.03 (0.86–1.07)	1.34 (1.21–1.47)	1.37 (1.24–1.51)	< 0.0001
All-cause mortality					
Cases, n (%)	575 (4.01)	594 (5.15)	983 (7.05)	1013 (8.50)	
Incidence rate per 1000 person-years	4.44 (4.09–4.82)	5.74 (5.30–6.22)	8.27 (7.77–8.81)	10.00 (9.41–10.60)	
Model 1	Reference	1.29 (1.15–1.45)	1.85 (1.67–2.05)	2.24 (2.02–2.48)	< 0.0001
Model 2	Reference	1.15 (1.03–1.29)	1.26 (1.13–1.40)	1.36 (1.22–1.51)	< 0.0001
Model 3	Reference	1.07 (0.95–1.20)	1.24 (1.11–1.38)	1.28 (1.15–1.43)	< 0.0001

Model 1: unadjusted

Model 2: adjusted for age and sex

Model 3: further adjusted for education, income, physical activity, smoking status, drinking status, history of hypertension, diabetes, dyslipidemia, antihypertensive agents, antidiabetic agents, lipid-lowering agents, body mass index, systolic blood pressure, diastolic blood pressure, total cholesterol, estimated glomerular filtration rate, and high sensitivity C reactive protein

The median value of cumTyG was 34.44 × year

In the subtype analyses of CVD, similar results were yielded for stroke, ischemic stroke, myocardial infarction, and heart failure, with the highest HRs in the group of a cumTyG ≥ median and a TyG slope < 0. However, most of the HRs in hemorrhagic stroke and atrial fibrillation were not significantly significant, which could be limited by the relatively small sample size of cases (Additional file 1: Figure S3; Table S4). The sensitivity analyses using competing risk model (Additional file 1: Table S5), excluding the events within the first two years (n = 50,652, Additional file 1: Table S6), excluding participants who were treated with antidiabetic or lipid-lowering agents (n = 46,686, Additional file 1: Table S7), or excluding participants with abnormal levels of FBG or TG (n = 33,758, Additional file 1: Table S8) all generated similar findings with the primary analysis.

### Subgroup analysis

**Fig. 5:**
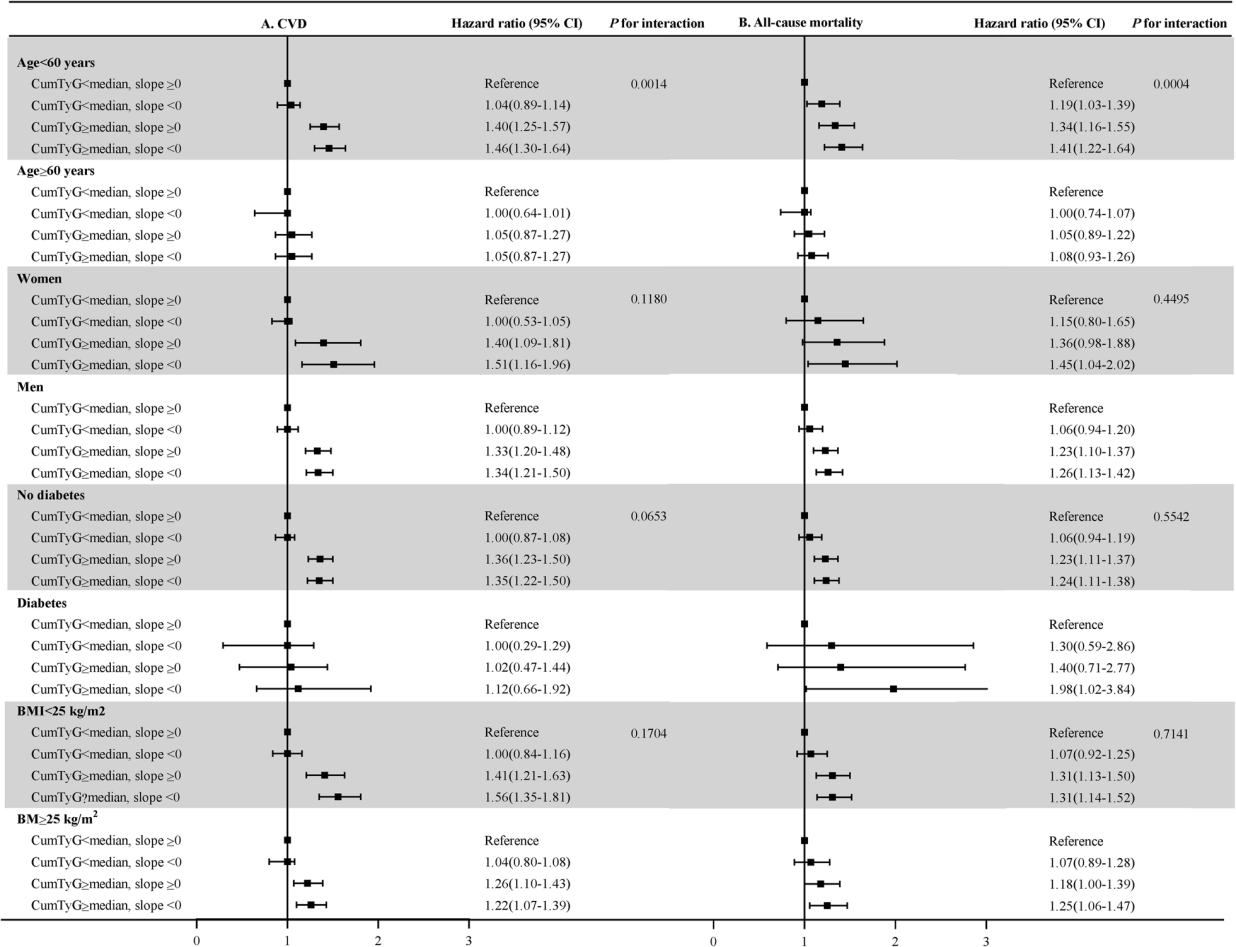
Subgroup analyses for the association of the combination of cumTyG and time course of TyG index accumulation with the risk of cardiovascular disease and all-cause mortality. *BMI* body mass index,* CI* confidence interval,* CVD* cardiovascular disease,* cumTyG* cumulative triglyceride glucose index,* TyG index* triglyceride glucose index. Adjusted for age, sex, education, income, drinking status, smoking status, physical activity, history of hypertension, diabetes, dyslipidemia, antihypertensive agents, antidiabetic agents, lipid-lowering agents, body mass index, systolic blood pressure, diastolic blood pressure, fasting blood glucose, total cholesterol, estimated glomerular filtration rate, and high sensitivity C-reactive protein other than variables for stratification

Results of subgroup analyses are presented in Fig. 5. Generally, participants in the group of a cumTyG ≥ median and a TyG slope < 0 had a higher risk of CVD and all-cause mortality across various subgroups. There was a significant interaction between age and the group in relation to the risk of CVD (*P* for interaction = 0.0014) and all-cause mortality (*P* for interaction = 0.0004), the hazards of the combination between a cumTyG ≥ median and a TyG slope < 0 on CVD and all-cause mortality were more prominent in young adults (age < 60 years; HR was 1.46 [95% CI, 1.30–1.64 ] for CVD and 1.41 [95% CI, 1.22–1.64] for all-cause mortality) than that in older adults (age ≥ 60 years; HR was 1.05 [95% CI, 0.87–1.27 ] for CVD and 1.08 [95% CI, 0.93–1.26] for all-cause mortality).

## Discussion

This study showed that the risk of future incident CVD and all-cause mortality was associated with the combination of the overall cumTyG exposure and time course of TyG accumulation. Specifically, our data suggest that cumTyG accumulated early confers a greater risk than when the same cumTyG is accumulated later. The findings underscored the importance of controlling an optimal level of TyG index earlier in life, because lower TyG later, even when low enough to result in the same overall accumulation, does not fully reverse risk acquired earlier. However, it is important to recognize that these data do not in any way suggest that there is no primary prevention benefit to lowering TyG index no matter when elevated TyG index lowering is start; the data only suggest apparently persistent increase in later CVD and mortality risk conferred by high TyG index levels experienced early in life. Furthermore, young adults with a cumTyG over median and a negative slope of TyG time course had a greater risk of subsequent incident CVD and all-cause mortality then older adults.

Previous studies regarding on the longitudinal associations of TyG index with CVD and all-cause mortality were mainly focused on exposure amount of the TyG index. For instance, in the studies of Cui et al. and Wang et al., cumTyG index was categorized into quartiles, and the results showed that a higher cumTyG index was associated with increasing risk of CVD and stroke in the general population. Although the studies demonstrated that a longer duration of a high TyG index exposure was associated with a higher risk of CVD, whether an early accumulation or later accumulation conferred a higher risk was not investigated, and the true time course of cumTyG index accumulation was not taken into account in these studies.

Our study filled the knowledge gap by demonstrating that time course of cumTyG accumulation modulated the associations of cumTyG and risk of CVD with all-cause mortality. Specifically, individuals with the same cumulative exposure to TyG but with a greater fraction of the exposure occurring earlier in life had the greatest risk of incident CVD and all-cause mortality. That means the same cumTyG index exposure accumulated earlier contributes more to the future CVD and all-cause mortality risk compared with later in life. The influence of the time course of TyG index accumulation with incident CVD and all-cause mortality may be inferred from data derived from primary prevention trials of IR. A systematic review and meta-analysis included nine clinical trials showed that in patients with IR or diabetes, pioglitazone had beneficial effects in reducing the risk of major adverse cardiovascular events, and the benefits were more with the treatment start early and long [30]. The Action to Control Cardiovascular Risk in Diabetes (ACCORD) randomized clinical trial showed early intensive as opposed to standard glycemic control CVD in type 2 diabetes could prevent CVD [31]. These data suggested that prolonged exposure to lower IR, beginning early in life, is contributed more to the risk reduction of CVD.

Additionally, our subgroup analysis showed that the association of a higher cumTyG exposure combined with a decreased trend of TyG time course with CVD and all-cause mortality was more prominent in young adults. In line with our findings, a prospective cohort of US female health professionals participating in the Women’s Health Study showed that IR was a strong risk factor for premature onset of coronary heart disease, and associations that attenuated with increasing age at onset [32]. A pooled analysis of population-based studies of Framingham Heart Study, Prevention of Renal and Vascular End-stage Disease Study, and Multi-Ethnic Study of Atherosclerosis demonstrated that the attributable risk of diabetes and dyslipidemia on the risk of CVD was greater among young participants [33]. Our findings, taken together with other studies, highlighted the importance of IR control started earlier and across the life course.

The mechanisms underlying the associations of time course of TyG index accumulation with the development of CVD and all-cause mortality remains uncertain, but several possibilities have been proposed. First, the TyG index was the product of FBG and TG, it has been noted that FBG mainly reflects IR in the liver, whereas TG mainly reflects IR in adipocytes [34]. Thus, it can be postulated that the TyG index may reflected IR affecting both organs. Second, IR is associated with chronic inflammation, oxidative stress, and the endothelial dysfunction, all these may contribute to the progression of atherosclerosis, and eventually lead to the occurrence the CVD and affect the longevity of individual’s life [35–37]. Participants with a higher cumTyG accumulated earlier at life were more likely to experience a longer time course of vascular and organ damage, thus may increase the risk of CVD and all-cause mortality. Finally, participants with a higher cumTyG accumulation and a negative TyG slope ended to have more severe and complex diseases, such as hypertension, diabetes, and dyslipidemia, which are risk factors of CVD and all-cause mortality.

There are some interesting implications of this study, particularly when viewed in the context of other studies. The first is that assessment of risk of future CVD and all-cause mortality is informed by considering not just the total amount of TyG index accumulation, but also the time course of TyG index accumulation. Using the data from the Kailuan study, we developed a risk model that takes into account both of these descriptors of longitudinal TyG index exposure. In current practice, the TyG index at the time is used without trying to incorporate the modulation of that risk by the time course of the patient’s TyG index level. The results presented here both emphasized the dependence of risk, not just on the present TyG index level, but also TyG index versus time course, and offer a model to quantify the modulation of risk by the time course of TyG index. Finally, these data suggested that lowering TyG index should start early, or even in young adults, which may show a major reduction in CVD incidence and mortality compared to risk reduction started later.

The strengths of the study included the large sample size with a long follow-up, and incorporation both the cumTyG amount and time course of TyG accumulation into a single risk parameter to predict future CVD and mortality. However, several limitations should also be noted. First, owing to a shortage of records insulin concentration data, we could not compare the predictive value of cumTyG index time course with those of HOMA-IR and the hyperinsulinaemic euglycaemic clamp test for the development of CVD and mortality. Second, the sex distribution of the sample was unbalanced. However, the associations of time course of cumTyG accumulation with CVD and all-cause mortality were statistically robust, given that a significant interaction was not identified when data were stratified according to sex. Third, although other potential cardiac risk factors were adjusted, we still cannot exclude the possibility of residual or unassessed confounders due to the observational nature of the present analysis.

## Conclusion

Incident CVD event risk depends on total cumTyG index and the time course of cumTyG accumulation. The same cumTyG accumulated earlier, compared with later in life, resulted in a greater risk increase, which emphasized the importance of optimal TyG index control starting early in life.

## Supplementary Information

**Additional file 1: Table S1**. Baseline characteristics between included and excluded participants.** Table S2**. Baseline characteristics according to slope of the TyG index.** Table S3**. Association of time course of cumTyG accumulation with risk of CVD subtypes.** Table S4**. Association of cumulative accumulation and slope of the TyG index with CVD subtypes.** Table S5**. Sensitivity analysis by using competing risk model or excluding CVD event during the follow-up.** Table S6**. Sensitivity analysis by using 2-lagged analysis (n = 50652).** Table S7**. Sensitivity analysis by excluding participants were treated with antidiabetic or lipid-lowering agents (n = 46686).** Table S8**. Sensitivity analysis by excluding participants with abnormal FBG or TG levels (n = 33758).** Figure S1**. The flowchart of the study.** Figure S2**. Cumulative TyG index and TyG slope calculated across 3 examinations in 1 participants. Average TyG index between consecutive examinations as A1 and A2. Cumulative TyG index was calculated as (A1 × time06–08 + A2 × time08–10), showed by the dotted area, × year.** Figure S3**. Kaplan-Meier curve of cardiovascular disease subtypes incidence rate by the combination of cumTyG and time course of TyG index accumulation.

## Data Availability

The datasets used and/or analyzed during the current study are available from the corresponding author on reasonable request.
