# Supplementary material for: Time course of the triglyceride glucose index accumulation with the risk of cardiovascular disease and all-cause mortality
Source: Cardiovasc Diabetol. 2022 Sep 13;21:183. doi: 10.1186/s12933-022-01617-2 (PMC9472367; doi:10.1186/s12933-022-01617-2)
Supplement: Supplementary file 1 — Additional file 1: Table S1. Baseline characteristics between included and excluded participants. Table S2. Baseline characteristics according to slope of the TyG index. Table S3. Association of time course of cumTyG accumulation with risk of CVD subtypes. Table S4. Association of cumulative accumulation and slope of the TyG index with CVD subtypes. Table S5. Sensitivity analysis by using competing risk model or excluding CVD event during the follow-up. Table S6. Sensitivity analysis by using 2-lagged analysis (n = 50652). Table S7. Sensitivity analysis by excluding participants were treated with antidiabetic or lipid-lowering agents (n = 46686). Table S8. Sensitivity analysis by excluding participants with abnormal FBG or TG levels (n = 33758). Figure S1. The flowchart of the study. Figure S2. Cumulative TyG index and TyG slope calculated across 3 examinations in 1 participants. Average TyG index between consecutive examinations as A1 and A2. Cumulative TyG index was calculated as (A1 × time06–08 + A2 × time08–10), showed by the dotted area, × year. Figure S3. Kaplan-Meier curve of cardiovascular disease subtypes incidence rate by the combination of cumTyG and time course of TyG index accumulation. [file 12933_2022_1617_MOESM1_ESM.docx]

**Supplemental Material**

Table S1. Baseline characteristics between included and excluded participants

| Characteristics | Excluded (N=49776) | Included  (N=51734) | *P* value |
| --- | --- | --- | --- |
| Age, years | 55.23±12.73 | 48.76±11.77 | <0.0001 |
| Men, n (%) | 41723 (83.82) | 39387 (76.13) | <0.0001 |
| High school or above, n (%) | 2583 (5.43) | 4187 (8.38) | <0.0001 |
| Income≥1000RMB, n (%) | 6264 (13.18) | 7751 (15.53) | <0.0001 |
| Current smoker, n (%) | 16319 (34.03) | 17476 (34.72) | 0.0230 |
| Current alcohol, n (%) | 16409 (34.21) | 20243 (40.20) | <0.0001 |
| Active physical activity, n (%) | 8302 (16.68) | 6979 (13.49) | <0.0001 |
| Hypertension, n (%) | 25003 (50.23) | 19650 (37.98) | <0.0001 |
| Diabetes mellitus, n (%) | 5445 (10.94) | 4044 (7.82) | <0.0001 |
| Dyslipidemia, n (%) | 18921 (38.01) | 17472 (33.77) | <0.0001 |
| Antihypertensive agents, n (%) | 7003 (14.07) | 4311 (8.33) | <0.0001 |
| Hypoglycemic agents, n (%) | 1528 (3.07) | 953 (1.84) | <0.0001 |
| Lipid-lowering agents, n (%) | 559 (1.12) | 404 (0.78) | <0.0001 |
| Body mass index, kg/m^2^ | 25.05±3.51 | 25.04±3.48 | 0.8223 |
| SBP, mmHg | 134.41±22 | 127.9±19.64 | <0.0001 |
| DBP, mmHg | 84.67±12.17 | 82.37±11.29 | <0.0001 |
| FBG, mmol/L | 5.58±1.85 | 5.38±1.52 | <0.0001 |
| Total cholesterol, mmol/L | 4.99±1.17 | 4.91±1.12 | <0.0001 |
| Triglyceride, mmol/L | 1.68±1.38 | 1.67±1.37 | 0.2803 |
| LDL cholesterol, mmol/L | 2.41±0.93 | 2.29±0.90 | <0.0001 |
| HDL cholesterol, mmol/L | 1.54±0.41 | 1.55±0.39 | <0.0001 |
| eGFR, mL/min/1.73m^2^ | 79.37±25.96 | 84.31±25.11 | <0.0001 |
| hs-CRP, mg/L | 2.53±6.42 | 2.30±6.51 | <0.0001 |

Abbreviations: DBP, diastolic blood pressure; eGFR, estimated glomerular filtration rate; FBG, fasting blood glucose; hs-CRP, high-sensitivity C-reactive protein; LDL, low density lipoprotein; HDL, high density lipoprotein; SBP, systolic blood pressure.

Table S2. Baseline characteristics according to slope of the TyG index

| Characteristics | Slope < 0  (N=23450) | Slope≥0  (N=28284) | *P* value |
| --- | --- | --- | --- |
| Age, years | 49.86±11.61 | 47.85±11.82 | <0.0001 |
| Men, n (%) | 18100 (77.19) | 21287 (75.26) | <0.0001 |
| High school or above, n (%) | 1738 (7.41) | 2449 (8.66) | <0.0001 |
| Income≥1000RMB, n (%) | 3228 (13.77) | 4523 (15.99) | <0.0001 |
| Current smoker, n (%) | 7547 (32.18) | 9929 (35.10) | <0.0001 |
| Current alcohol, n (%) | 8715 (37.16) | 11528 (40.76) | <0.0001 |
| Active physical activity, n (%) | 3271 (13.95) | 3708 (13.11) | 0.0054 |
| Hypertension, n (%) | 10031 (42.78) | 9619 (34.01) | <0.0001 |
| Diabetes mellitus, n (%) | 2559 (10.91) | 1485 (5.25) | <0.0001 |
| Dyslipidemia, n (%) | 9867 (42.08) | 7605 (26.89) | <0.0001 |
| Antihypertensive agents, n (%) | 2044 (8.72) | 2267 (8.02) | 0.0041 |
| Hypoglycemic agents, n (%) | 521 (2.22) | 432 (1.53) | <0.0001 |
| Lipid-lowering agents, n (%) | 176 (0.75) | 228 (0.81) | 0.4747 |
| Body mass index, kg/m^2^ | 25.25±3.48 | 24.86±3.46 | <0.0001 |
| SBP, mmHg | 129.90±20.00 | 126.27±19.11 | <0.0001 |
| DBP, mmHg | 83.45±11.44 | 81.48±11.06 | <0.0001 |
| FBG, mmol/L | 5.64±1.83 | 5.17±1.15 | <0.0001 |
| Total cholesterol, mmol/L | 4.91±1.24 | 4.92±1.02 | 0.2472 |
| Triglyceride, mmol/L | 2.10±1.63 | 1.32±0.97 | <0.0001 |
| LDL cholesterol, mmol/L | 2.32±0.89 | 2.27±0.90 | <0.0001 |
| HDL cholesterol, mmol/L | 1.57±0.39 | 1.54±0.39 | <0.0001 |
| eGFR, mL/min/1.73m^2^ | 81.21±24.84 | 86.88±25.01 | <0.0001 |
| hs-CRP, mg/L | 2.17±5.78 | 2.40±6.95 | 0.0001 |
| CumTyG | 35.14±4.77 | 35.04±4.67 | 0.0128 |
| Slope, year^-1^ | -0.11±0.10 | 0.12±0.10 | <0.0001 |

Abbreviations: cumTyG, cumulative TyG index; DBP, diastolic blood pressure; eGFR, estimated glomerular filtration rate; FBG, fasting blood glucose; hs-CRP, high-sensitivity C-reactive protein; LDL, low density lipoprotein; HDL, high density lipoprotein; SBP, systolic blood pressure; TyG index, triglyceride glucose index.

Table S3. Association of time course of cumTyG accumulation with risk of CVD subtypes

|  | Slope^a^ | | |  | cumTyG06-08 | |  | cumTyG08-10^b^ | |
| --- | --- | --- | --- | --- | --- | --- | --- | --- | --- |
|  | <0 | ≥0 | *P* value |  | HR (95% CI) | *P* value |  | HR (95% CI) | *P* value |
| Stroke |  |  |  |  |  |  |  |  |  |
| Cases, n (%) | 1083 (4.62) | 1157 (4.09) |  |  |  |  |  |  |  |
| Incidence rate per  1000 person-years | 5.39(5.07-5.72) | 4.73(4.47-5.01) |  |  |  |  |  |  |  |
| Model 1 | 1.14(1.05-1.24) | Reference | 0.0020 |  | 1.04(1.03-1.05) | <0.0001 |  | 1.03(0.99-1.05) | 0.0932 |
| Model 2 | 1.13(1.04-1.22) | Reference | 0.0045 |  | 1.04(1.04-1.05) | <0.0001 |  | 1.03(0.98-1.04) | 0.1219 |
| Model 3 | 1.09(1.01-1.19) | Reference | 0.0324 |  | 1.03(1.02-1.04) | <0.0001 |  | 1.02(0.97-1.02) | 0.3489 |
| Ischemic stroke |  |  |  |  |  |  |  |  |  |
| Cases, n (%) | 963 (4.11) | 1026 (3.63) |  |  |  |  |  |  |  |
| Incidence rate per  1000 person-years | 4.78(4.49-5.09) | 4.19(3.94-4.45) |  |  |  |  |  |  |  |
| Model 1 | 1.14(1.05-1.25) | Reference | 0.0029 |  | 1.05(1.04-1.06) | <0.0001 |  | 1.03(0.98-1.05) | 0.1132 |
| Model 2 | 1.13(1.04-1.23) | Reference | 0.0063 |  | 1.05(1.04-1.06) | <0.0001 |  | 1.02(0.98-1.03) | 0.1879 |
| Model 3 | 1.10(1.01-1.20) | Reference | 0.0350 |  | 1.04(1.02-1.04) | <0.0001 |  | 1.02(0.97-1.02) | 0.3988 |
| Hemorrhagic stroke |  |  |  |  |  |  |  |  |  |
| Cases, n (%) | 117 (0.50) | 140 (0.49) |  |  |  |  |  |  |  |
| Incidence rate per  1000 person-years | 0.57(0.48-0.69) | 0.56(0.48-0.67) |  |  |  |  |  |  |  |
| Model 1 | 1.01(0.79-1.30) | Reference | 0.9098 |  | 1.01(0.98-1.04) | 0.4002 |  | 1.02(0.98-1.06) | 0.3046 |
| Model 2 | 1.00(0.79-1.28) | Reference | 0.9750 |  | 1.01(0.98-1.04) | 0.3469 |  | 1.01(0.97-1.05) | 0.5608 |
| Model 3 | 0.96(0.75-1.23) | Reference | 0.7511 |  | 1.00(0.97-1.03) | 0.9758 |  | 1.00(0.96-1.04) | 0.9124 |
| Myocardial infarction |  |  |  |  |  |  |  |  |  |
| Cases, n (%) | 277 (1.18) | 267 (0.94) |  |  |  |  |  |  |  |
| Incidence rate per  1000 person-years | 1.36(1.21-1.53) | 1.08(0.96-1.22) |  |  |  |  |  |  |  |
| Model 1 | 1.26(1.06-1.49) | Reference | 0.0071 |  | 1.06(1.04-1.08) | <0.0001 |  | 1.05(0.98-1.07) | 0.3268 |
| Model 2 | 1.24(1.05-1.47) | Reference | 0.0120 |  | 1.06(1.04-1.08) | <0.0001 |  | 1.05(0.98-1.06) | 0.4673 |
| Model 3 | 1.20(1.01-1.42) | Reference | 0.0339 |  | 1.05(1.03-1.06) | <0.0001 |  | 1.03(0.95-1.03) | 0.8631 |
| Heart failure |  |  |  |  |  |  |  |  |  |
| Cases, n (%) | 379 (1.62) | 395 (1.40) |  |  |  |  |  |  |  |
| Incidence rate per  1000 person-years | 1.86(1.69-2.06) | 1.60(1.45-1.77) |  |  |  |  |  |  |  |
| Model 1 | 1.17(1.04-1.34) | Reference | 0.0325 |  | 1.07(1.05-1.08) | <0.0001 |  | 1.04(1.00-1.05) | 0.0471 |
| Model 2 | 1.16(1.02-1.34) | Reference | 0.0370 |  | 1.07(1.05-1.07) | <0.0001 |  | 1.03(0.98-1.07) | 0.0982 |
| Model 3 | 1.11(1.00-1.28) | Reference | 0.0467 |  | 1.05(1.03-1.06) | <0.0001 |  | 1.02(0.96-1.05) | 0.1276 |
| Atrial fibrillation |  |  |  |  |  |  |  |  |  |
| Cases, n (%) | 168 (0.72) | 153 (0.54) |  |  |  |  |  |  |  |
| Incidence rate per  1000 person-years | 0.82(0.71-0.96) | 0.62(0.53-0.72) |  |  |  |  |  |  |  |
| Model 1 | 1.33(1.07-1.66) | Reference | 0.0103 |  | 1.08(1.06-1.10) | <0.0001 |  | 1.03(0.93-1.05) | 0.3473 |
| Model 2 | 1.32(1.06-1.64) | Reference | 0.0127 |  | 1.08(1.06-1.09) | <0.0001 |  | 1.02(0.93-1.04) | 0.3897 |
| Model 3 | 1.26(1.01-1.56) | Reference | 0.0424 |  | 1.06(1.04-1.08) | <0.0001 |  | 1.00(0.90-1.02) | 0.7854 |

Abbreviations: CI, confidence interval; CVD, cardiovascular disease; cumTyG, cumulative triglyceride glucose index; HR, hazard ratio

Model 1: unadjusted;

Model 2: adjusted for age and sex;

Model 3: further adjusted for education, income, physical activity, smoking status, drinking status, history of hypertension, diabetes, dyslipidemia, antihypertensive agents, antidiabetic agents, lipid-lowering agents, body mass index, systolic blood pressure, diastolic blood pressure, total cholesterol, estimated glomerular filtration rate, and high sensitivity C reactive protein.

^a^ further adjusted for mean TyG during 2006-2010.

^b^ further adjusted for cumulative TyG during 2006-2008.

Table S4. Association of cumulative accumulation and slope of the TyG index with CVD subtypes

| Outcomes | CumTyG<median, slope ≥ 0 | CumTyG<median, slope < 0 | CumTyG≥median, slope ≥ 0 | CumTyG≥ median, slope < 0 | *P* for trend |
| --- | --- | --- | --- | --- | --- |
| Stroke |  |  |  |  |  |
| Cases, n (%) | 447 (3.12) | 394 (3.42) | 710 (5.09) | 689 (5.78) |  |
| Incidence rate per  1000 person-years | 3.49(3.18-3.83) | 3.86(3.49-4.26) | 6.10(5.66-6.56) | 6.96(6.46-7.50) |  |
| Model 1 | Reference | 1.11(0.97-1.27) | 1.81(1.60-2.03) | 2.06(1.83-2.32) | <0.0001 |
| Model 2 | Reference | 1.06(0.93-1.22) | 1.58(1.40-1.78) | 1.71(1.52-1.94) | <0.0001 |
| Model 3 | Reference | 1.03(0.83-1.09) | 1.39(1.23-1.57) | 1.41(1.24-1.59) | <0.0001 |
| Ischemic stroke |  |  |  |  |  |
| Cases, n (%) | 385 (2.68) | 344 (2.98) | 641 (4.60) | 619 (5.19) |  |
| Incidence rate per  1000 person-years | 3.00(2.72-3.32) | 3.36(3.03-3.74) | 5.49(5.08-5.94) | 6.24(5.77-6.75) |  |
| Model 1 | Reference | 1.12(0.97-1.30) | 1.90(1.68-2.16) | 2.16(1.90-2.45) | <0.0001 |
| Model 2 | Reference | 1.08(0.93-1.25) | 1.67(1.47-1.90) | 1.80(1.58-2.05) | <0.0001 |
| Model 3 | Reference | 1.04(0.84-1.12) | 1.46(1.29-1.67) | 1.48(1.29-1.69) | <0.0001 |
| Hemorrhagic stroke |  |  |  |  |  |
| Cases, n (%) | 62 (0.43) | 52 (0.45) | 78 (0.56) | 65 (0.55) |  |
| Incidence rate per  1000 person-years | 0.48(0.37-0.62) | 0.50(0.38-0.66) | 0.66(0.53-0.82) | 0.64(0.50-0.82) |  |
| Model 1 | Reference | 1.05(0.73-1.52) | 1.39(1.00-1.95) | 1.36(0.96-1.93) | 0.0310 |
| Model 2 | Reference | 1.01(0.70-1.46) | 1.21(0.86-1.70) | 1.12(0.79-1.60) | 0.3657 |
| Model 3 | Reference | 1.00(0.61-1.27) | 1.02(0.64-1.32) | 1.07(0.76-1.51) | 0.9080 |
| Myocardial infarction |  |  |  |  |  |
| Cases, n (%) | 99 (0.69) | 102 (0.88) | 168 (1.20) | 175 (1.47) |  |
| Incidence rate per  1000 person-years | 0.77(0.63-0.93) | 0.99(0.82-1.20) | 1.42(1.22-1.65) | 1.74(1.50-2.02) |  |
| Model 1 | Reference | 1.29(0.98-1.70) | 1.86(1.45-2.38) | 2.27(1.78-2.91) | <0.0001 |
| Model 2 | Reference | 1.23(0.93-1.63) | 1.62(1.26-2.08) | 1.87(1.45-2.40) | <0.0001 |
| Model 3 | Reference | 1.10(0.83-1.45) | 1.42(1.10-1.83) | 1.53(1.19-1.98) | <0.0001 |
| Heart failure |  |  |  |  |  |
| Cases, n (%) | 126 (0.88) | 123 (1.07) | 269 (1.93) | 256 (2.15) |  |
| Incidence rate per  1000 person-years | 0.98(0.82-1.16) | 1.20(1.00-1.43) | 2.28(2.03-2.57) | 2.55(2.26-2.88) |  |
| Model 1 | Reference | 1.22(0.95-1.57) | 2.24(1.81-2.77) | 2.52(2.03-3.11) | <0.0001 |
| Model 2 | Reference | 1.13(0.88-1.45) | 1.70(1.37-2.10) | 1.75(1.41-2.18) | <0.0001 |
| Model 3 | Reference | 1.01(0.79-1.30) | 1.35(1.08-1.69) | 1.43(1.15-1.78) | 0.0010 |
| Atrial fibrillation |  |  |  |  |  |
| Cases, n (%) | 61 (0.43) | 61 (0.53) | 92 (0.66) | 107 (0.90) |  |
| Incidence rate per  1000 person-years | 0.47(0.37-0.61) | 0.59(0.46-0.76) | 0.78(0.63-0.95) | 1.06(0.88-1.28) |  |
| Model 1 | Reference | 1.25(0.88-1.78) | 1.57(1.14-2.17) | 2.15(1.57-2.94) | <0.0001 |
| Model 2 | Reference | 1.13(0.79-1.61) | 1.11(0.80-1.54) | 1.37(0.99-1.90) | 0.0646 |
| Model 3 | Reference | 1.04(0.73-1.49) | 1.03(0.73-1.43) | 1.20(0.86-1.67) | 0.2791 |

Abbreviations: CVD, cardiovascular disease; cumTyG, cumulative triglyceride glucose index

Model 1: unadjusted;

Model 2: adjusted for age and sex;

Model 3: further adjusted for education, income, physical activity, smoking status, drinking status, history of hypertension, diabetes, dyslipidemia, antihypertensive agents, antidiabetic agents, lipid-lowering agents, body mass index, systolic blood pressure, diastolic blood pressure, total cholesterol, estimated glomerular filtration rate, and high sensitivity C reactive protein.

The median value of cumTyG was 34.44×year.

Table S5. Sensitivity analysis by using competing risk model or excluding CVD event during the follow-up

| Outcomes | CumTyG<median, slope ≥ 0 | CumTyG<median, slope < 0 | CumTyG≥median, slope ≥ 0 | CumTyG≥ median, slope < 0 | *P* for trend |
| --- | --- | --- | --- | --- | --- |
| CVD | Reference | 1.02(0.86-1.07) | 1.33(1.20-1.46) | 1.35(1.22-1.49) | <0.0001 |
| Stroke | Reference | 1.02(0.83-1.09) | 1.32(1.21-1.54) | 1.36(1.22-1.57) | <0.0001 |
| Ischemic stroke | Reference | 1.02(0.84-1.12) | 1.32(1.20-1.64) | 1.35(1.27-1.66) | <0.0001 |
| Hemorrhagic stroke | Reference | 1.00(0.60-1.27) | 1.06(0.75-1.50) | 1.07(0.63-1.31) | 0.6064 |
| Myocardial infarction | Reference | 1.10(0.83-1.45) | 1.41(1.08-1.83) | 1.52(1.16-1.98) | <0.0001 |
| Heart failure | Reference | 1.01(0.79-1.30) | 1.34(1.07-1.68) | 1.42(1.14-1.77)) | <0.0001 |
| Atrial fibrillation | Reference | 1.02(0.72-1.51) | 1.04(0.72-1.51) | 1.19(0.84-1.70) | 0.3351 |
| All-cause mortality* | Reference | 1.09(0.96-1.24) | 1.26(1.12-1.42) | 1.28(1.14-1.45) | <0.0001 |

Abbreviations: CVD, cardiovascular disease; cumTyG, cumulative triglyceride glucose index

Adjusted for age, sex, education, income, physical activity, smoking status, drinking status, history of hypertension, diabetes, dyslipidemia, antihypertensive agents, antidiabetic agents, lipid-lowering agents, body mass index, systolic blood pressure, diastolic blood pressure, total cholesterol, estimated glomerular filtration rate, and high sensitivity C reactive protein.

The median value of cumTyG was 34.44×year.

* Sensitivity analysis was performed by excluding CVD events during the follow-up period rather than competing risk model.

Table S6. Sensitivity analysis by using 2-lagged analysis (n=50652)

| Outcomes | CumTyG<median, slope ≥ 0 | CumTyG<median, slope < 0 | CumTyG≥median, slope ≥ 0 | CumTyG≥ median, slope < 0 | *P* for trend |
| --- | --- | --- | --- | --- | --- |
| CVD | Reference | 1.03(0.85-1.08) | 1.37(1.23-1.52) | 1.35(1.21-1.51) | <0.0001 |
| Stroke | Reference | 1.04(0.82-1.09) | 1.39(1.22-1.58) | 1.36(1.19-1.55) | <0.0001 |
| Ischemic stroke | Reference | 1.04(0.81-1.11) | 1.47(1.28-1.69) | 1.43(1.24-1.65) | <0.0001 |
| Hemorrhagic stroke | Reference | 0.93(0.62-1.40) | 1.02(0.59-1.34) | 1.15(0.79-1.68) | 0.8225 |
| Myocardial infarction | Reference | 1.08(0.79-1.48) | 1.38(1.03-1.85) | 1.47(1.09-1.97) | 0.0036 |
| Heart failure | Reference | 1.04(0.71-1.25) | 1.33(1.03-1.71) | 1.51(1.18-1.93) | 0.0024 |
| Atrial fibrillation | Reference | 1.09(0.70-1.71) | 1.09(0.72-1.67) | 1.45(0.97-2.19) | 0.0617 |
| All-cause mortality | Reference | 1.07(0.94-1.22) | 1.33(1.18-1.49) | 1.28(1.14-1.45) | <0.0001 |

Abbreviations: CVD, cardiovascular disease; cumTyG, cumulative triglyceride glucose index

Adjusted for age, sex, education, income, physical activity, smoking status, drinking status, history of hypertension, diabetes, dyslipidemia, antihypertensive agents, antidiabetic agents, lipid-lowering agents, body mass index, systolic blood pressure, diastolic blood pressure, total cholesterol, estimated glomerular filtration rate, and high sensitivity C reactive protein.

The median value of cumTyG was 34.44×year.

Table S7. Sensitivity analysis by excluding participants were treated with antidiabetic or lipid-lowering agents (n=46686)

| Outcomes | CumTyG<median, slope ≥ 0 | CumTyG<median, slope < 0 | CumTyG≥median, slope ≥ 0 | CumTyG≥ median, slope < 0 | *P* for trend |
| --- | --- | --- | --- | --- | --- |
| CVD | Reference | 1.06(0.87-1.10) | 1.41(1.27-1.57) | 1.38(1.24-1.54) | <0.0001 |
| Stroke | Reference | 1.05(0.83-1.11) | 1.41(1.24-1.61) | 1.38(1.20-1.58) | <0.0001 |
| Ischemic stroke | Reference | 1.05(0.83-1.14) | 1.50(1.30-1.73) | 1.46(1.26-1.68) | <0.0001 |
| Hemorrhagic stroke | Reference | 0.91(0.62-1.33) | 1.01(0.67-1.45) | 1.03(0.71-1.50) | 0.8809 |
| Myocardial infarction | Reference | 1.17(0.87-1.59) | 1.68(1.27-2.22) | 1.70(1.28-2.25) | <0.0001 |
| Heart failure | Reference | 1.07(0.81-1.40) | 1.57(1.23-2.00) | 1.32(1.02-1.70) | 0.0045 |
| Atrial fibrillation | Reference | 1.06(0.72-1.56) | 1.12(0.77-1.62) | 1.43(1.00-2.04) | 0.0420 |
| All-cause mortality | Reference | 1.06(0.94-1.20) | 1.27(1.13-1.42) | 1.29(1.15-1.45) | <0.0001 |

Abbreviations: CVD, cardiovascular disease; cumTyG, cumulative triglyceride glucose index

Adjusted for age, sex, education, income, physical activity, smoking status, drinking status, history of hypertension, diabetes, dyslipidemia, antihypertensive agents, antidiabetic agents, lipid-lowering agents, body mass index, systolic blood pressure, diastolic blood pressure, total cholesterol, estimated glomerular filtration rate, and high sensitivity C reactive protein.

The median value of cumTyG was 34.44×year.

Table S8. Sensitivity analysis by excluding participants with abnormal FBG or TG levels (n=33758)

| Outcomes | CumTyG<median, slope ≥ 0 | CumTyG<median, slope < 0 | CumTyG≥median, slope ≥ 0 | CumTyG≥ median, slope < 0 | *P* for trend |
| --- | --- | --- | --- | --- | --- |
| CVD | Reference | 1.05(0.76-1.09) | 1.26(1.12-1.41) | 1.30(1.09-1.44) | <0.0001 |
| Stroke | Reference | 1.02(0.74-1.04) | 1.26(1.09-1.46) | 1.35(1.14-1.61) | <0.0001 |
| Ischemic stroke | Reference | 1.03(0.77-1.10) | 1.33(1.14-1.55) | 1.41(1.17-1.70) | <0.0001 |
| Hemorrhagic stroke | Reference | 0.98(0.44-1.13) | 1.00(0.64-1.46) | 1.05(0.58-1.61) | 0.9763 |
| Myocardial infarction | Reference | 1.06(0.49-1.15) | 1.12(0.76-1.65) | 1.23(0.90-1.68) | 0.2109 |
| Heart failure | Reference | 1.05(0.70-1.28) | 1.19(0.87-1.63) | 1.51(1.17-1.94) | 0.0197 |
| Atrial fibrillation | Reference | 1.07(0.74-1.55) | 1.09(0.73-1.62) | 1.18(0.77-1.80) | 0.5014 |
| All-cause mortality | Reference | 1.03(0.90-1.18) | 1.21(1.07-1.37) | 1.25(1.09-1.44) | <0.0001 |

Abbreviations: CVD, cardiovascular disease; cumTyG, cumulative triglyceride glucose index; FBG, fasting blood glucose; TG, triglyceride.

Adjusted for age, sex, education, income, physical activity, smoking status, drinking status, history of hypertension, diabetes, dyslipidemia, antihypertensive agents, antidiabetic agents, lipid-lowering agents, body mass index, systolic blood pressure, diastolic blood pressure, total cholesterol, estimated glomerular filtration rate, and high sensitivity C reactive protein.

The median value of cumTyG was 34.4×year.


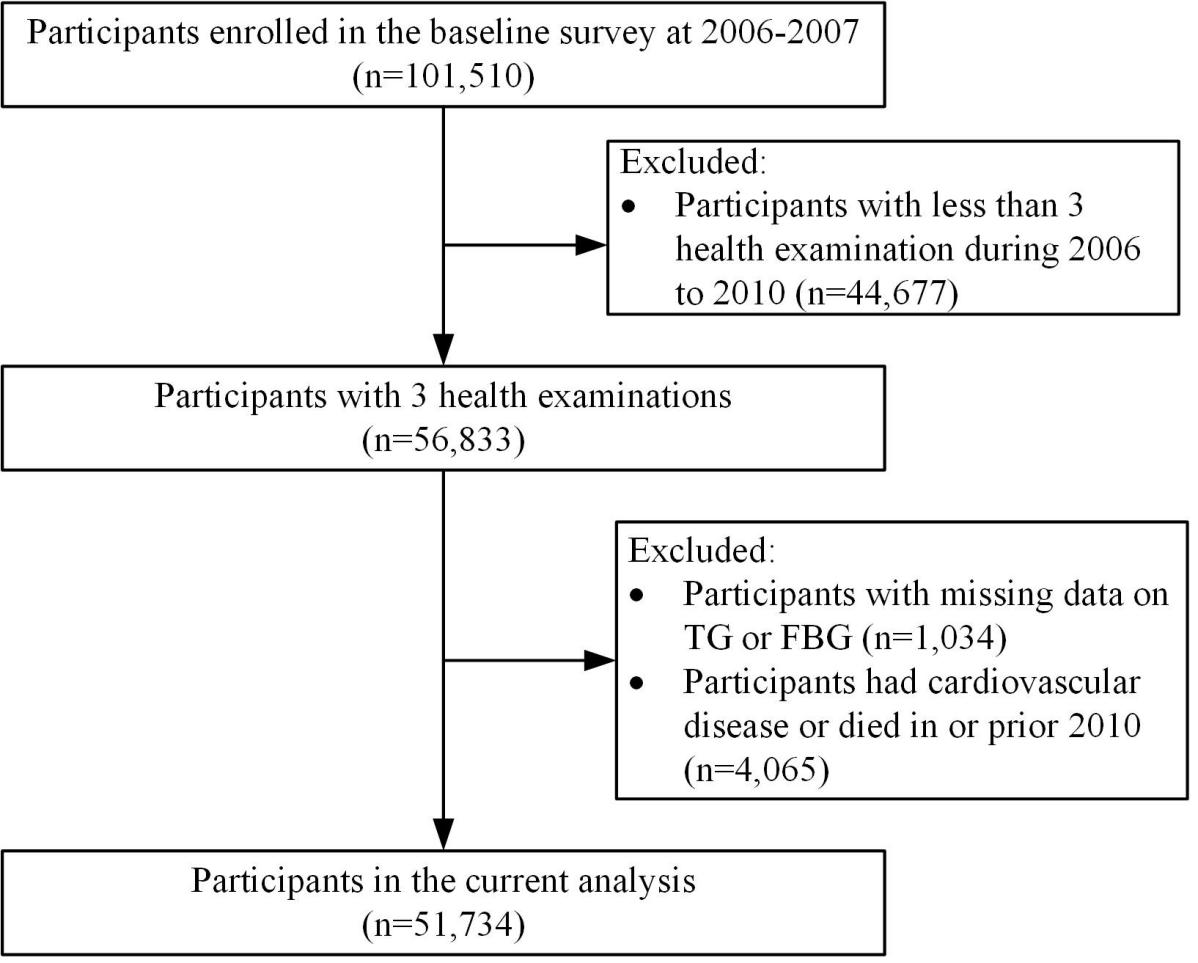


**Figure S1. The flowchart of the study**

Abbreviations: FBG, fasting blood glucose; TG, triglyceride


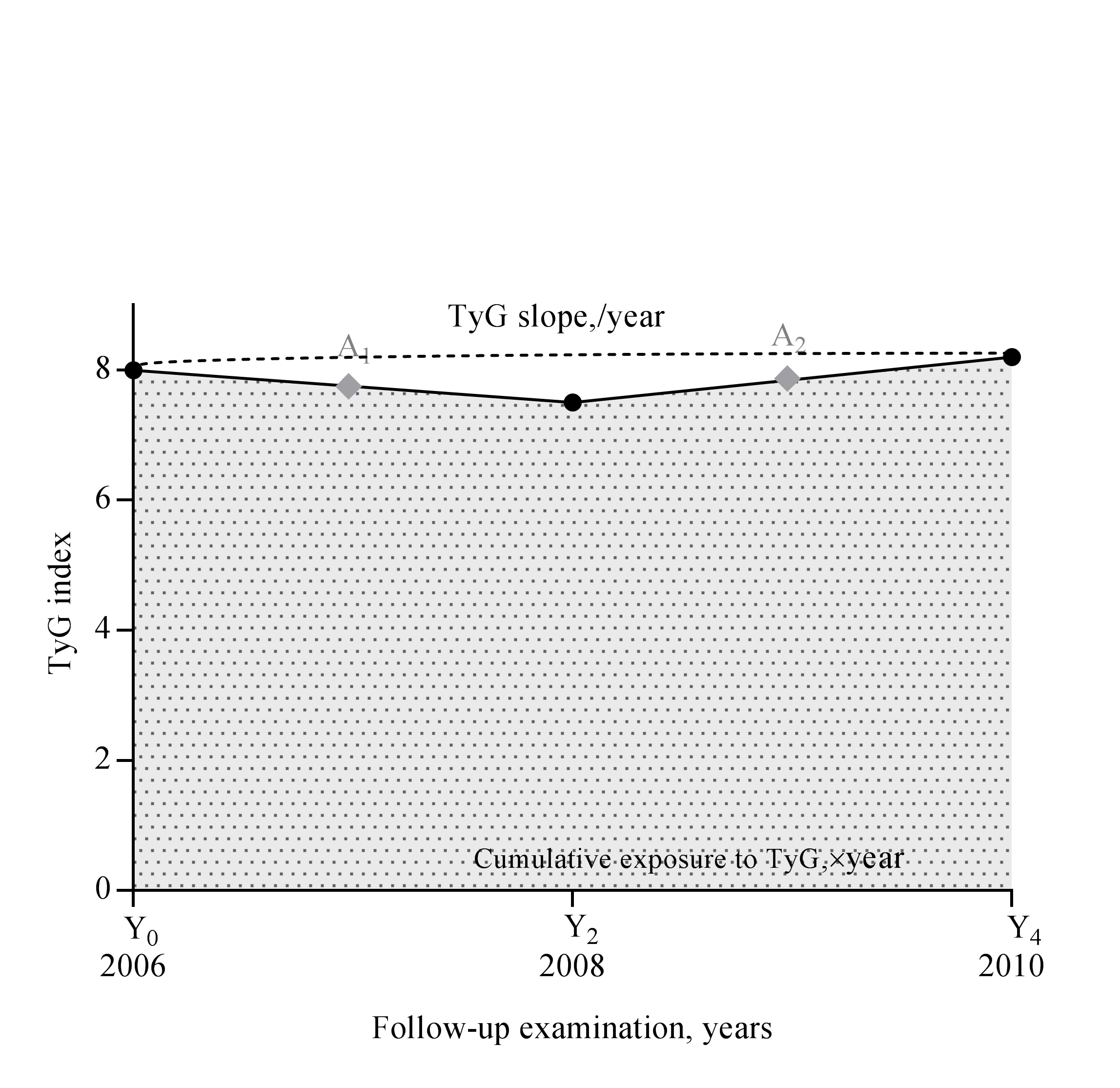


**Figure S2. Cumulative TyG index and TyG slope calculated across 3 examinations in 1 participants. Average TyG index between consecutive examinations as A1 and A2. Cumulative TyG index was calculated as (A_1_ × time_06–08_ + A_2_ × time_08–10_), showed by the dotted area, × year.**

TyG slope was obtained using a linear regression, TyG index values at follow-up visits were used to calculate the TyG index.

Abbreviation: TyG index, triglyceride glucose index.


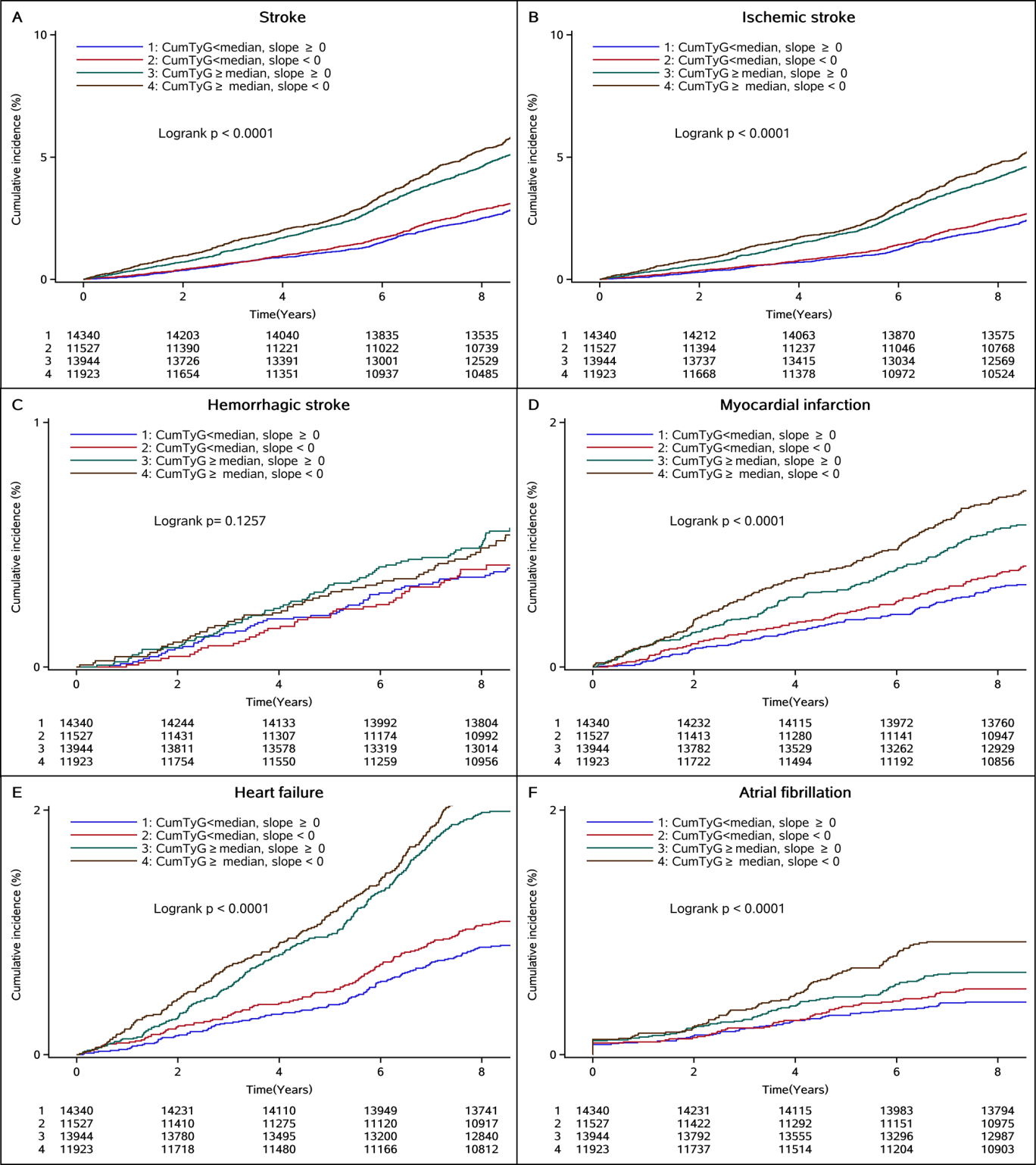


**Figure S3. Kaplan-Meier curve of cardiovascular disease subtypes incidence rate by the combination of cumTyG and time course of TyG index accumulation.**

Abbreviations: cumTyG, cumulative triglyceride glucose index; TyG index, triglyceride glucose index.
